# Editorial: *Chlamydia trachomatis* infection: Epidemiology, prevention, clinical, and basic science research

**DOI:** 10.3389/fpubh.2023.1167690

**Published:** 2023-03-07

**Authors:** Changchang Li, Jason Ong, Weiming Tang, Cheng Wang

**Affiliations:** ^1^Dermatology Hospital of Southern Medical University, Guangzhou, China; ^2^Melbourne Sexual Health Centre, Monash University, Melbourne, VIC, Australia

**Keywords:** review, epidemiology, prevention, clinical, chlamydia

*Chlamydia trachomatis* (*C. trachomatis*) infection is a global health concern due to its serious reproductive health consequences, such as pelvic inflammatory disease (PID), ectopic pregnancy, and tubal infertility (1). It also facilitates HIV infection and transmission (2). In 2020, WHO estimated nearly 129 million new cases of *C. trachomatis* infection worldwide each year (3). Early detection and treatment have been implemented in high-income countries (HICs) for many years, potentially reducing the incidence of PID and tubal infertility (4). However, routine screening has been lacking in low- and middle-income countries (LMICs). The appropriate screening strategy remains unclear due to a lack of evidence on epidemic patterns, the cost-effectiveness of screening for *C. trachomatis* infection, and the different socioeconomic contexts in these regions. In this issue of Research Topic, “*Chlamydia trachomatis infection: Epidemiology, prevention, clinical, and basic science research*,” the authors reported epidemic characteristics, the cost-effectiveness of screening, and improved treatment (Hu et al.; Wang et al.; Shi et al.; Pérez-González et al.; Sun et al.; Montes-Olivas et al.; Ning et al.; Liu et al.; Weng et al.; Yu et al.; Xu et al.; Huston et al.).

Although the high incidence and morbidity rates of *C*. *trachomatis* infection among women of reproductive age have been reported, there are few studies on the epidemic in this population in LMICs. Three studies from China reported a high prevalence of *C*. *trachomatis* infection of 9, 6, and 4, respectively, among 8,324 patients attending STI clinics, 306 men who have sex with men (MSM), and 3,580 female sex workers (FSWs) (Wang et al.; Hu et al.; Shi et al.). Consistently, all three studies found that individuals aged < 25 years, those with low levels of education, and those who had ever or currently had sexually transmitted infections (STIs) were more likely to be infected with *C*. *trachomatis*. In addition, a cohort study in northwestern Spain suggested that PrEP users have a higher risk of *C*. *trachomatis* infection, especially among individuals who engage in chemsex (Pérez-González et al.). Further, a study from China reported that pre-pregnant couples should also be given more attention due to the higher risk of adverse pregnancy outcomes in *C*. *trachomatis*-infected women (32%, 9/28) and men (25%, 4/16) (Sun et al.). The above studies highlight the need for targeted interventions (e.g., routine screening) in pre-pregnant couples and key populations, including attendees of STI clinics, MSM, and FSWs, especially those aged < 25 years, with low education and who have ever or currently had STIs.

Early detection and timely treatment are effective interventions to prevent reproductive harm from *C*. *trachomatis* infection. Routine *C*. *trachomatis* infection screening has been implemented in the UK, USA, and some European countries. However, the cost-effectiveness of screening is controversial, and the optimal screening strategy for each key population is poorly understood. A modeling study compared the cost-effectiveness of universal screening and targeted screening in the high-risk population of Hong Kong, China (Montes-Olivas et al.). It found that the most effective strategy was targeted screening with contact tracing for individuals with multiple partners. The ICER for targeted screening with contact tracing at 20 and 40% effectiveness was $4,634, and $7,219 per QALY gained, respectively (10-year time horizon). Before the screening, the clinical symptom may provide clues to identify the target population and improve the intervention strategy (Ning et al.). The responding symptoms include frequent urination/urgency/urodynia/itching, balanitis, and inguinal lymph node enlargement in men, and vaginal secretion increase or odors and vaginal itching in women, respectively. For example, another study found that *C. trachomatis* genotype H may be a sign for the target population because of its higher risk of cervical intraepithelial lesions (Liu et al.). The location of taking biospecimen is one of the important determinants of screening effectiveness.

Regarding the positivity rate, the rectal specimen is more appropriate for MSM (Weng et al.). A study in China found that the prevalence of *C. trachomatis* infection using rectal samples was almost six times higher than the prevalence in urine among MSM. Still, only 44% of MSM accepted, and 96% (128/133) of them successfully provided a valid rectal specimen.

Clinical treatment of *C*. *trachomatis* infection is considered convenient and safe, but the development of antibiotic-resistant strains and other treatment failures are often observed in patients. A study in China found that Rhein (4, 5-dihydroxyanthraquinone-2-carboxylic acid, a monomer primarily extracted from rhubarb) could improve the treatment of *C*. *trachomatis* infection (Yu et al.). Experiments *in vitro* and *in vivo* showed that Rhein could inhibit the growth of *C*. *trachomatis* by regulating pathogen-host cell interactions and synergizing the inhibitory effect of azithromycin (Xu et al.). Besides treating *C*. *trachomatis* infection cases, current guidelines recommend patient-delivered partner therapy to reduce chlamydia re-infection. Meanwhile, a study from Hong Kong examined the impact of screening only and screening plus accelerated partner therapy and showed that accelerated partner therapy did not significantly affect overall chlamydia prevalence and caused overtreatment (Montes-Olivas et al.).

Re-testing for chlamydia 3 months after treatment to detect possible re-infection has been recommended in HICs. However, re-testing does not appear to be universally implemented. A study of 5,806 heterosexuals with chlamydia in Melbourne, Australia, showed that only 36% were re-tested for chlamydia within 1 year, and 15% were reinfected (Xu et al.). Another cohort study of 305 women with urogenital chlamydial infection showed that 12% had recurrent infection after treatment with azithromycin and that recurrent infection was associated with sexual contact (Huston et al.). The low rate of chlamydia re-testing and the high rate of chlamydia re-infection highlight the need to optimize partner management and encourage testing for re-infection at 3 months.

Compared to primary infection, chlamydia DNA load was higher in women who experienced recurrent infection (Huston et al.). Vaginal chlamydial gene expression (ompA, euo, omcB, htrA, trpAB) was significantly higher at the time of recurrent infection or repeated positive tests during follow-up compared to baseline; two of the selected immune genes analyzed and had significantly lower expression at the time of recurrent infection (Huston et al.). The results suggest that repeat infections with chlamydia may be more transcriptionally active at certain genes after azithromycin treatment. There may be immunological changes after treatment that interact with repeat exposures to establish active infection. Therefore, more regular testing of women at the highest risk is needed to reduce the risk of sequelae.

Chlamydia is a significant threat to the population's sexual and reproductive health. The high prevalence and impact on reproductive health by the studies reported in this issue underscore the need for targeted CT screening of high-risk populations. Given differences in epidemiology and socioeconomic contexts, more research on the cost-effectiveness evaluation of routine screening, case management, and basic science is needed to improve prevention and clinical strategies.

## Author contributions

CL drafted the first manuscript, other co-editors provided comments, and prepared the final version, which all co-authors approved. CW, JO, and WT contributed to formulating key lessons. All authors contributed to the article and approved the submitted version.
