# Corrigendum: Editorial: *Chlamydia trachomatis* infection: epidemiology, prevention, clinical, and basic science research

**DOI:** 10.3389/fpubh.2024.1467902

**Published:** 2024-08-14

**Authors:** Changchang Li, Jason Ong, Weiming Tang, Cheng Wang

**Affiliations:** ^1^Dermatology Hospital of Southern Medical University, Guangzhou, China; ^2^Melbourne Sexual Health Centre, Monash University, Melbourne, VIC, Australia

**Keywords:** review, epidemiology, prevention, clinical, chlamydia

In the published article, there was an error in the Funding statement. The funder and grant number were not included in the Funding Information Statement of the original article. The correct Funding statement appears below:

